# Assessment of piRNA biogenesis and function in testicular germ cell tumors and their precursor germ cell neoplasia *in situ*

**DOI:** 10.1186/s12885-017-3945-6

**Published:** 2018-01-04

**Authors:** Ildar V. Gainetdinov, Yulia V. Skvortsova, Sofia A. Kondratieva, Alexey Klimov, Alexey A. Tryakin, Tatyana L. Azhikina

**Affiliations:** 1 Department of Genomics and Postgenomic Technologies, Shemyakin-Ovchinnikov Institute of Bioorganic Chemistry, Russian Academy of Sciences, Moscow, Russia 117997; 2 Department of Oncology, Blokhin Russian Cancer Research Center, Moscow, Russia; 3 Department of Clinical Pharmacology and Chemotherapy, Blokhin Russian Cancer Research Center, Moscow, Russia

**Keywords:** PIWI, piRNA, Testicular germ cell tumor, Seminoma, Nonseminoma, GCNIS, Germ cell neoplasia in situ, Germ cell cancer

## Abstract

**Background:**

Aberrant overexpression of PIWI/piRNA pathway proteins is shown for many types of tumors. Interestingly, these proteins are downregulated in testicular germ cell tumors (TGCTs) compared to normal testis tissues. Here, we used germline and TGCT markers to assess the piRNA biogenesis and function in TGCTs and their precursor germ cell neoplasia in situ (GCNIS).

**Methods:**

We used small RNA deep sequencing, qRT-PCR, and mining public RNAseq/small RNA-seq datasets to examine PIWI/piRNA gene expression and piRNA biogenesis at four stages of TGCT development: (i) germ cells in healthy testis tissues, (ii) germ cells in testis tissues adjacent to TGCTs, (iii) GCNIS cells and (iv) TGCT cells. To this end, we studied three types of samples: (a) healthy testis, (b) testis tissues adjacent to two types of TGCTs (seminomas and nonseminomas) and containing both germ cells and GCNIS cells, as well as (c) matching TGCT samples.

**Results:**

Based on our analyses of small RNA-seq data as well as the presence/absence of expression correlation between PIWI/piRNA pathway genes and germline or TGCT markers, we can suggest that piRNA biogenesis is intact in germ cells present in healthy adult testes, and adjacent to TGCTs. Conversely, GCNIS and TGCT cells were found to lack PIWI/piRNA pathway gene expression and germline-like piRNA biogenesis. However, using an *in vitro* cell line model, we revealed a possible role for a short PIWIL2/HILI isoform expressed in TGCTs in posttranscriptional regulation of the youngest members of LINE and SINE classes of transposable elements. Importantly, this regulation is also implemented without involvement of germline-like biogenesis of piRNAs.

**Conclusions:**

Though further studies are warranted, these findings suggest that the conventional germline-like PIWI/piRNA pathway is lost in transition from germ cells to GCNIS cells.

**Electronic supplementary material:**

The online version of this article (10.1186/s12885-017-3945-6) contains supplementary material, which is available to authorized users.

## Background

Testicular germ cell tumors (TGCTs) are the most frequent malignancies among male adolescents and young adults, with rising incidence [1, 2]. They are very heterogeneous cancers that are histologically classified into seminomas and nonseminomas [3]. The common origin of TGCTs is believed to be a precursor lesion called germ cell neoplasia in situ (GCNIS) [4], which is formed from primordial germ cells or gonocytes following the arrest of germ cell differentiation [5]. Although there has been a number of comprehensive genome-wide association studies, very few biologically relevant mutations were found [6]. Moreover, the increasing bulk of research suggests a more prominent role for the epigenetic causes of TGCTs [7].

One of the key players in spermatogenesis is the PIWI/piRNA pathway responsible for suppression of transposon expression [8] and post-transcriptional regulation of several genes essential for normal spermatogenesis [9, 10]. Deregulated expression of PIWI orthologs in human TGCTs was first detected in seminomas [11]. Possible involvement of piRNA pathway deregulation in development of TGCTs was previously probed. Specifically, Ferreira *et al* found a correlation between hypermethylation of PIWI gene promoters and loss of their expression in TGCTs compared to normal testis of healthy individuals [12]. This group also demonstrated a concomitant hypomethylation of L1 retrotransposons in TGCTs [12]. Another study by Rounge et al presented deep sequencing data of small RNAs for a set of normal testis, GCNIS samples and TGCTs, and stated that the loss of piRNAs is a hallmark of TGCT samples [13].

Earlier, our group attempted to study transformation from normal germ cells to a TGCT by incorporating matched GCNIS cell samples into analysis. We revealed that, compared to normal testis, expression of PIWI proteins was significantly lower in testis samples adjacent to seminomas but only slightly decreased in those adjacent to nonseminomas [14]. This observation can arise from two possible settings. Firstly, the PIWI/piRNA pathway might be specifically silenced in the course of development of seminomas since its expression is lost in tissues adjacent to this type of TGCTs. Alternatively, this can be explained by the fact that testis tissues adjacent to TGCTs contain both GCNIS cells and germ cells. Here, in order to distinguish between these two possibilities, we assessed correlation of expression between PIWI/piRNA pathway genes and either germline or TGCT markers in healthy testis (containing only germ cells) and testis tissues adjacent to TGCTs (containing both germ cells and GCNIS cells). This approach also allowed us to examine four stages of neoplastic transformation using three types of samples: (i) normal germ cells (in healthy testis tissues), (ii) germ cells and (iii) GCNIS cells adjacent to TGCTs (in testis samples adjacent to TGCTs) and (iv) TGCT cells (in TGCT samples). Additionally, we employed small RNA deep sequencing and elaborate bioinformatic pipeline to study piRNA biogenesis at these four stages in detail. Finally, we used an *in vitro* cell line model to reveal the role of PIWIL2/HILI short isoform (PL2L60A) expressed in TGCTs/GCNIS in regulating TE expression posttranscriptionally.

## Methods

### Tissue collection

Twenty-one pairs of TGCT tissues and corresponding adjacent normal testicular parenchyma were obtained from orchiectomy specimens: 7 seminomas and 14 nonseminomas. 4 samples of normal testis tissue were obtained from prostate cancer patients undergoing surgical castration. The samples were immediately frozen in liquid nitrogen. All patients provided written informed consent according to the federal law, and the study was approved by the ethical committees of the Shemyakin-Ovchinnikov Institute of Bioorganic Chemistry of the Russian Academy of Sciences and Blokhin Russian Cancer Research Center after reviewing patients’ consent and information forms.

### RNA extraction, gene expression assays and small RNA libraries preparation

Total RNA extraction and purification was performed with Trizol reagent (Thermo Fisher Scientific, USA). cDNA synthesis was performed with MintReverse Transcriptase and following qPCRs with qPCRmix-HS SYBR system (Evrogen, Russia) on Lightcycler 480 (Roche, Switzerland) according to the manufacturers’ instructions. Primer pairs used in amplification are listed in

Additional file 1: Table S8 and reactions were run for 40 cycles of 95°C – 20 sec, 60°C – 20 sec, 72°C – 20 sec. The assays were performed in triplicates and results analyzed in LinRegPCR v 2014.6.

Small RNA libraries were generated from 2mkg of total RNA with TruSeq Small RNA Library Preparation Kit (Illumina, USA) according to manufacturer’s instructions and sequenced on HiSeq 2000 Sequencing System to generate 50nt long reads. Raw sequencing reads were deposited in SRA as bioproject PRJNA352412 (https://www.ncbi.nlm.nih.gov/bioproject/?term=PRJNA352412, Additional file 1: Table S9). Publicly available datasets used in this study are listed in Supplementary Additional file 1: Table S9.

### Small RNA datasets analysis

Raw reads were quality filtered and trimmed with FASTX-Toolkit (http://hannonlab.cshl.edu/fastx_toolkit/). Bowtie v1.0.0 [15] was used to align trimmed reads to hg38 Human Genome Assembly with zero mismatches. Alignment procedure was based on Tailor aligner [16]. Briefly, unaligned reads were trimmed by one nucleotide from 3’ end and realigned again with zero mismatches allowed. This step was repeated two more times and only aligned reads (four alignments in total: after 0/1/2/3 nucleotides trimmed from 3’end) were kept for further analyses.

Integrative Genomics Viewer v2.3.74 [17] was used to visualize alignment data. Statistical calculations were performed with R custom scripts or GraphPad Prism v5.00. Miscellaneous data processing was conducted with custom Bash/Awk/Perl scripts available on request.

Assessment of small RNA coverage of transposable elements was done with the approach described earlier in Pezic *et al* [18]. Briefly, up to 10,000 genomic hits mapped with zero mismatches were reported for each read and 1/N score was assigned for each genomic hit found, where N is the total number of all possible genomic hits for a read. Further, scores for all genomic hits were summed by transposable element subfamily.

Alignments to transposable element consensi were performed with two mismatches allowed and were used to calculate TE-specific ping-pong Z-score and distribution of reads along the consensi.

Probabilistic piRNA cluster calling was done with TBr2_duster.pl/sRNAmapper.pl/ proTRAC_2.1.2.pl pipeline by Rosenkranz and Zischler [19, 20] with proTRAC parameters as follows: -pdens 0.05 -1Tor10A 0.1 -1Tand10A 0.1 -clstrand 0.5 -clsplit 0.05. Finally, a list of 1299 of human piRNA clusters from meta-analysis by Chirn *et al*. [21] was used to assess overlap with the probabilistically identified clusters.

### GTEx RNAseq data for adult testis samples and publicly available RNAseq datasets from fetal human testis samples

Expression data quantified in RPKM were downloaded from http://gtexportal.org/ and normalized to *ACTB* expression. Publicly available RNA-seq libraries for fetal testis (Additional file 1: Table S9) were filtered with FASTX-Toolkit and a conventional TopHat v2.0.9/Cufflinks v2.1.1 pipeline with default parameters and reference annotation from Ensembl release 83 was employed [22–25].

### Cell lines and PIWIL2 knockdown experiment

Cell line TERA1 (ATCC HTB-105) was purchased from ATTC (USA) and maintained in DMEM/F12 (1:1) (Thermo Fisher Scientific, USA) supplemented with 10% FCS (Thermo Fisher Scientific, USA) [26]. For PIWIL2 knockdown experiment TERA1 was reverse transfected with custom synthesized siRNA duplexes (DNK Syntez, Russia) in presence of Lipofectamin RNAiMAX (Thermo Fisher Scientific, USA) as recommended by the manufacturer. Biological triplicates were used to ensure reproducibility. The sequences of siRNA duplexes were as follows: scrambled RNA -GCAUGAGCGACCACUCCUAdTdT and UAGGAGUGGUCGCUCAUGCdTdT, siRNA 1 - CCAUUGGCAGAACACGUCCdTdT and GGACGUGUUCUGCCAAUGGdTdT, siRNA 2 - CUUCCUUAACCCAGUUUAGdTdT and CUAAACUGGGUUAAGGAAGdTdT.

### Assessment of DNA methylation level

DNA was extracted with Wizard Genomic DNA Purification Kit (Promega, USA) and bisulfite conversion was done with EpiTect Bisulfite Kit (Qiagen, Netherlands). 258bp long genomic fragment around L1HS/L1PA2/L1PA3 promoter was PCR amplified with Q5 High-Fidelity DNA Polymerase (NEB, USA) using primers GGTTTATTTTATTAGGGAGTGTTAGATAG and AAAACCCTCTAAACCAAATATAAAATATA. Amplicons were converted into barcoded sequencing libraries with NEBNext DNA Library Prep Kit (NEB, USA) and sequenced on HiSeq 2000 Sequencing System (Illumina, USA) to generate 150nt long paired-end reads. Level of methylation was assessed as the mean for all CG sites in the amplified fragment. Biological and technical triplicates were used to ensure reproducibility.

### Chromatin immunoprecipitation (ChIP) assay

ChIP was performed as described earlier [27, 28] using the following antibodies to human histone modifications: H3K4me3 (ab8580, Abcam, UK) and H3K9me3 (ab8898, Abcam, UK). DNA was purified using QIAquick PCR Purification Kit (Qiagen, Netherlands). qPCR was performed using qPCRmix-HS SYBR system (Evrogen, Russia) on Lightcycler 480 (Roche, Switzerland) in accordance with the manufacturers’ instructions with primer pairs GGTTCATCTCACTAGGGAGTGCC and AGTGACCCGATTTTCCAGGTG for full-length L1HS/L1PA2/L1PA3, CAACATGGTGAAACCCCGTCTCT and GCCTCAGCCTCCCGAGTAG for all Alu subfamilies, ACCATCCCGGCTAAAACGGTGA and GCGATCTCGGCTCACTG for AluYa5. Reactions were run for 40 cycles of 95°C – 20 sec, 60°C – 20 sec, 72°C – 20 sec. Relative level of chromatin modification was quantified with “input control” serving as the reference. Biological and technical triplicates were used to ensure reproducibility and results were analyzed in LinRegPCR v 2014.6.

### L1 retrotransposition assay

Two biological replicates of retrotransposition assay were conducted with pJM101 plasmid in accordance with previously published protocol [29].

## Results

## Expression of piRNA biogenesis genes in germ cells in testis samples adjacent to TGCTs

To study the role of PIWI/piRNA pathway in development of TGCTs, we wanted to assess its function at four stages of neoplastic transformation using three types of samples: (i) normal germ cells (in healthy testis tissues), (ii) germ cells and (iii) GCNIS cells adjacent to TGCTs (in testis samples adjacent to TGCTs) and (iv) TGCT cells (in TGCT samples). Because testis samples adjacent to TGCTs contain both germ cells and GCNIS cells, we distinguished between those using correlation of piRNA pathway gene expression with either germline (*DDX4* and *DAZL* [30–32]) or TGCT markers (*NANOG* and *POU5F1* [33–37]). We also complemented our sample collection (4 normal adult testis and 21 matched pairs of GCNIS/TGCT: 7 seminomas and 14 nonseminomas) with 172 samples of normal testis available from GTEx project [38] as well as 5 fetal testis samples from public datasets.

Initially, we assessed the variability of gene expression in normal adult testis and found relative standard deviation to be as high as 74-110% (median: 92%) for the 14 PIWI/piRNA pathway genes. However, their expression is also highly correlated between each other and with the germline markers *DAZL* and *DDX4* (Pearson’s r=0.80-0.99, Additional file 1: Table S1), which may point to the fact that PIWI/piRNA pathway genes are expressed exclusively in germ cells in normal adult testis.

Surprisingly, in testis samples adjacent to TGCTs, the correlation of expression of the 14 piRNA biogenesis genes between each other and with germline markers is similarly high (Pearson’s r=0.81-0.99, Additional file 1: Table S2). Importantly, the correlation of expression of piRNA pathway genes with TGCT markers was found to be slightly negative (Additional file 1: Table S2). This finding could support the notion that PIWI/piRNA pathway genes are expressed only in germ cells present in the testis samples adjacent to TGCTs, but not in the GCNIS cells. Thus, the difference in expression of piRNA biogenesis genes can be largely determined by the extent of spermatogenesis taking place in these tissues (i.e., germ cell content), while GCNIS cells do not seem to express PIWI/piRNA pathway genes. Finally, as previously shown for three PIWI proteins [14] (Additional file 2: Figure S1), germline marker and PIWI/piRNA pathway gene expression was almost undetectable in TGCTs of both types unlike fetal testis.

### Small RNA profiling suggests normal piRNA biogenesis in germ cells present in testis tissues adjacent to TGCTs

Although piRNA biogenesis genes are expressed in germ cells present both in healthy testis tissues and in testis tissues adjacent to TGCTs, the function of the pathway could be altered in the latter. Germ cells adjacent to TGCTs might have acquired some properties of GCNIS or TGCT cells. In order to reveal any possible deregulation of the piRNA biogenesis in these germ cells, we generated small RNA libraries from normal testis, TGCTs as well as matched adjacent testis tissues. We also used publicly available datasets from both testis and somatic tissues [39]. As expected, expression levels of miRNA markers for GCNIS/TGCTs (miR-302/367/371-3 [33]) increase along neoplastic transformation axis from normal adult testis to TGCTs with testis tissues adjacent to TGCTs exhibiting intermediate level of expression of these markers (Additional file 2: Figure S2). Importantly, correlation between GCNIS/TGCT miRNA and gene markers (*POU5F1* and *NANOG*) is very high as well (Pearson’s r = 0.97-0.99), which is in agreement with the fact that both types of markers are expressed exclusively in GCNIS/TGCT cells.

Since testis tissues adjacent to nonseminomas exhibit expression level of germline markers similar to normal testis (Additional file 2: Figure S1), we expect these samples (GCNIS NS) to be representative of germ cells adjacent to TGCTs, while testis tissue samples adjacent to seminomas (GCNIS SE) as well as both types of TGCTs (NS and SE) are predicted to lack any piRNA biogenesis altogether (due to lack of germ cells).

**Fig. 1:**
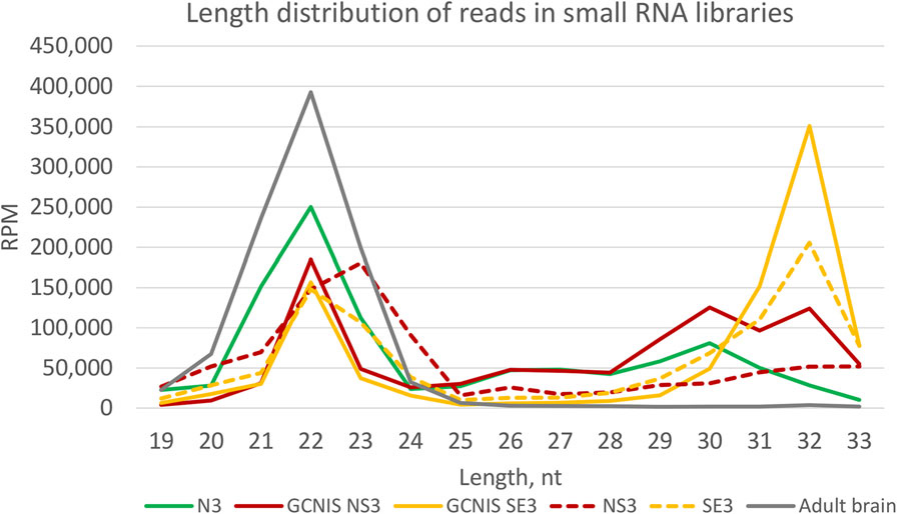
Length distribution of small RNA library reads in normal Adult testis (dataset N3), TGCTs (datasets SE3 for seminoma and NS3 for nonseminoma) and matched adjacent GCNIS-containing testis tissues (datasets GCNIS SE3 and GCNIS NS3, respectively) as well as somatic tissue (adult brain). The rest of the datasets are presented in Additional file 2: Figure S3

Indeed, only healthy adult testis and GCNIS NS samples exhibited presence of 26/30nt peaks (Fig. 1, Additional file 2: Figure S3), which suggests conventional germline-like piRNA biogenesis in germ cells adjacent to TGCTs. Of note, while inspecting length distribution of small RNA reads in various libraries, apart from 22nt peak corresponding to miRNAs, we could also observe 32nt peaks representing tRNA fragments (Fig. 1, Additional file 2: Figure S3).

**Fig. 2:**
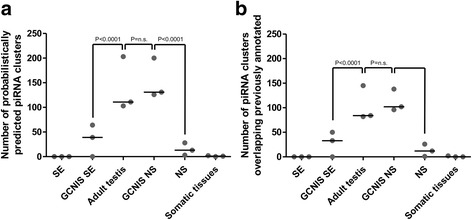
Number of piRNA clusters predicted from small RNA library reads using probabilistic approach (**a**) and number of probabilistically predicted piRNA clusters overlapping previously annotated ones (**b**). P-values for two-tailed Mann-Whitney test are presented (n.s. – non-significant)

To further look into PIWI/piRNA pathway function in germ cells adjacent to TGCTs, we studied other features of piRNA biogenesis in GCNIS NS (as the representative of germ cells adjacent to TGCTs). It is known that piRNAs in germ cells of adult mammals are produced from specific genomic loci termed piRNA-clusters [40]. Therefore, we evaluated the predicted number of these loci in each dataset using probabilistic approach developed by Rosenkranz *et al* [19]. Consistent with the presence of 26/30nt peaks, only germ cells in normal adult testis and GCNIS NS were predicted to produce piRNA from an appreciable number of piRNA clusters (Fig. 2A), whose coordinates overlapped with the previously published ones (Fig. 2B) [21]. All other datasets (TGCTs, GCNIS SE and somatic tissues) were predicted to produce piRNAs from significantly lower number of piRNA clusters.

Finally, since one of the established functions of piRNAs is repressing retrotransposons, we assessed the integrity of the pathway by looking at the fraction of TE-deriving small RNAs, ping-pong signature as well as first (1U) and tenth (10A) nucleotide biases (Additional file 2: Figure S4, Additional file 1: Tables S3-S5). We revealed the following relationship: the lower was the content of germ cells in a given sample the less prominent was the number of TE-deriving piRNAs, 1U bias, 10A bias, and Z-score for ping-pong signature. Essentially, PIWI/piRNA pathway features in germ cells present in GCNIS NS are identical to those in normal adult testis. Conversely, both TGCT and GCNIS SE samples lack signs of the germline-like PIWI/piRNA pathway and are similar to somatic tissues in this regard (Additional file 2: Table S4D-G).

Taken together, these data suggest that piRNA biogenesis in germ cells adjacent to TGCTs is intact and not altered.

### Short isoform of PIWIL2/HILI is able to repress TEs posttranscriptionally in TGCTs/GCNIS

One of the previously reported characteristics of TGCTs is expression of the short PIWIL2/HILI isoform (PL2L60A) [41]. Importantly, unlike full-length PIWIL2/HILI, PL2L60A lacks the part of its PIWI domain that is responsible for the slicing catalytical activity. It also does not possess N-terminal domain harboring conserved arginine residues that are methylated by PRMT5 and bound by TUDOR domain containing proteins, which is necessary for the recruitment of PIWIL2/HILI in PIWI/piRNA pathway complexes [42].

**Fig. 3:**
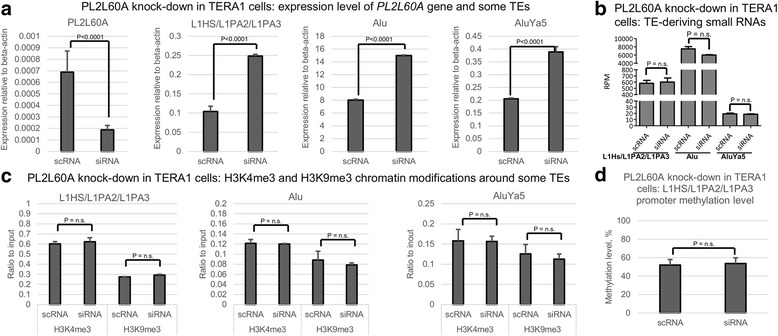
Knockdown of PL2L60A in TERA1 cell line leads to transcriptional upregulation of transposable elements. **a**, Transcription level of PL2L60A gene and the following retrotransposons: full-length L1HS/L1PA2/L1PA3s, all Alu elements, AluYa5 subfamily. **b**, Fraction of TE-deriving small RNAs for full-length L1HS/L1PA2/L1PA3s, all Alu elements, AluYa5 subfamily. **c**, Chromatin modifications (H3K4me3 and H3K9me3) around all Alu elements, AluYa5 subfamily, and 5’UTR promoter of full-length L1HS/L1PA2/L1PA3. **d**, DNA methylation level around 5’UTR promoter of full-length L1HS/L1PA2/L1PA3 TEs. The graphs show mean and standard deviation across three biological replicates. P-value for two-tailed Mann-Whitney test is presented (n.s. – non-significant). scRNA – control scrambled RNA, siRNA – anti-PL2L60A siRNA

We previously screened several cell lines for the expression of PIWIL2 isoforms and documented the presence of PL2L60A only in TERA1 among the TGCT-derived cell lines [41]. In order to probe if PL2L60A can be involved in TE targeting, we knocked down its expression in TERA1 with anti-PL2L60A siRNA and assessed expression change of TEs. We specifically chose to look at those TEs for which there is evidence of current active transposition in the human genome [43]. Among these are the youngest members of LINEs (L1HS/L1PA2/L1PA3, [44]) and SINEs (all Alu elements and AluYa5, in particular, [45]). We found that all these TE subfamilies were upregulated in TERA1 cell line after knockdown of PL2L60A (Fig. 3A). To test if this gain in their expression was accompanied by the change of the small RNA profile, we generated smallRNA-seq libraries from TERA1 cells treated with anti-PL2L60A siRNA and a scrambled control siRNA (scRNA). Analysis of these datasets failed to detect statistically significant changes of the level of the corresponding TE-derived small RNA fractions (Fig. 3B, Additional file 1: Table S6). Notably, small RNAs in TERA1 lack 1U and 10A biases, which is consistent with very low expression of *PLD6* in TERA1 cell line (data not shown) and presumably lost catalytical activity of PL2L60A, respectively (Additional file 1: Table S7). However, the length of these small RNAs was predominantly between 20-26nt allowing for a possibility that they can be generated with participation of PL2L60A as an RNA-binding protein (Additional file 1: Table S7).

To see whether this change of TE expression upon PL2L60A knock down is initiated transcriptionally or posttranscriptionally, we measured the level of DNA and H3K4me3/H3K9me3 histone methylation around promoters of L1HS/L1PA2/L1PA3 and Alu elements. Since we observed no significant change in these epigenetic marks (Fig. 3C&D), PL2L60A is likely to exert its influence over TE expression posttranscriptionally.

**Fig. 4:**
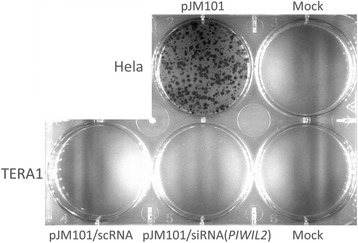
L1 retrotransposition assay performed with pJM101 vector on Hela (positive control) and TERA1 cell lines. The latter was treated either with siRNA for *PIWIL2* or with a scrambled RNA control

We also asked if the increased level of full-length L1Hs/L1PA2-4 transcripts could lead to activation of their retrotransposition. Using the assay developed by Moran *et al* [46], we found no evidence of L1 transposition in TERA1 (neither with normal PL2L60A expression nor after its knockdown, Fig. 4).

## Discussion

The PIWI family was initially discovered as proteins specifically expressed in testis and indispensable for germline development. Subsequently, they were found to be overexpressed in many types of cancers, suggesting their role in tumorigenesis [47]. On the contrary, in testicular germ cell tumors PIWI proteins were shown to be downregulated, which was correlated with their promoter hypermethylation and lack of piRNAs [12].

In this work, we attempted to follow changes of piRNA/PIWI pathway function along the “germ cell-GCNIS-TGCT” malignization axis at four stages: normal germ cells (in healthy testis tissues), germ cells adjacent to TGCTs and GCNIS cells (both in testis tissues adjacent to TGCTs) and TGCT cells (in TGCT samples). To distinguish between germ cells and GCNIS cells in the mixed sample of testis tissues adjacent to TGCTs, we used correlation of expression of PIWI/piRNA pathway genes with either germline or TGCT markers. Based on these correlations, we can argue that piRNA biogenesis genes are only expressed in normal germ cells present in either healthy adult testis or testis tissues adjacent to TGCTs. Moreover, piRNA biogenesis in germ cells residing next to TGCTs exhibits conventional features characteristic of germline piRNAs, which suggests that the PIWI/piRNA pathway is not altered in these germ cells. Conversely, neither GCNIS cells nor TGCT cells appear to express PIWI/piRNA pathway genes. GCNIS and TGCT cells also do not display piRNA biogenesis and their small RNA profiles are very similar to somatic tissues. Taken together, we can propose that the PIWI/piRNA pathway is essentially lost in transition from a normal germ cell to a GCNIS cell and is unlikely to be an oncogenic driver of TGCT development. Finally, although in most cases PIWI proteins have been shown to be overexpressed and possess oncogenic features [47–49], it is also possible that the PIWI/piRNA pathway can exert a tumor suppressor role in this setting and its loss might be one of the causes of germ cell transformation into GCNIS/TGCT.

The germline and TGCT markers that we used in this study are considered to be widely accepted “gold standards” both at mRNA and protein levels [31–33]. However, the sole use of correlation of expression of the PIWI/piRNA pathway with these markers still leaves room for alternative explanations of our results (for instance, that PIWI/piRNA pathway may function to a different extent in both germ cells and GCNIS cells in testis tissues adjacent to TGCTs). In order to unequivocally ascertain the boundaries of PIWI/piRNA pathway expression and piRNA biogenesis, further studies are warranted using either immunohistochemistry based approaches or elaborate cell sorting methods.

Finally, PIWIL2/HILI (one of the central players of PIWI/piRNA pathway) is expressed in TGCTs as a short isoform (PL2L60A). Therefore, we used TERA1 cell line model to interrogate whether this short isoform is involved in repression of TEs. Results of *in vitro* experiments suggest that PL2L60A may participate in TE silencing posttranscriptionally and in a slicing-independent manner. Analysis of small RNAs in TERA1 cells also indicates that the mechanism of this suppression does not involve conventional germline-like piRNA biogenesis.

## Conclusion

We examined the integrity of PIWI/piRNA pathway function at four stages of TGCT development: germ cells in healthy adult testes, germ cells adjacent to TGCTs, GCNIS cells and TGCT cells. Based on correlation with germline or TGCT marker expression, no evidence of its deregulation in germ cells adjacent to TGCTs was found, arguing against its role as an oncogenic driver of TGCT development. However, *in vitro* experiments demonstrated a possible role of the PIWIL2/HILI short isoform in repressing TEs, which could provide a growth advantage for TGCTs through securing their genome integrity.

## Additional files

Additional file 1: Tables S1-S9. (XLSX 101 kb)

Additional file 2: Figures S1-S4. (PDF 440 kb)

## Abbreviations

TGCT: Testicular germ cell tumor
GCNIS: Germ cell neoplasia in situ
piRNAs: PIWI-interacting RNAs
GCNIS SE/NS: Germ cell neoplasia in situ containing testis tissue adjacent to seminoma/nonseminoma, respectively
PGCs: Primordial germ cells
