# Supplementary material for: Assessment of piRNA biogenesis and function in testicular germ cell tumors and their precursor germ cell neoplasia in situ
Source: BMC Cancer. 2018 Jan 4;18:20. doi: 10.1186/s12885-017-3945-6 (PMC5755174; doi:10.1186/s12885-017-3945-6)

Supplementary figure S1. Expression level (qRT-PCR) of piRNA pathway genes in 21 pairs of testicular germ cell tumors and their precursors GCNIS for 7 seminomas (SE) and 14 nonseminomas (NS). The charts also show RNA-seq data from publicly available datasets from fetal and adult healthy testis tissues (GTEx and supplementary table S9). P-values for two-tailed Mann-Whitney test are presented (n.s. – non-significant).

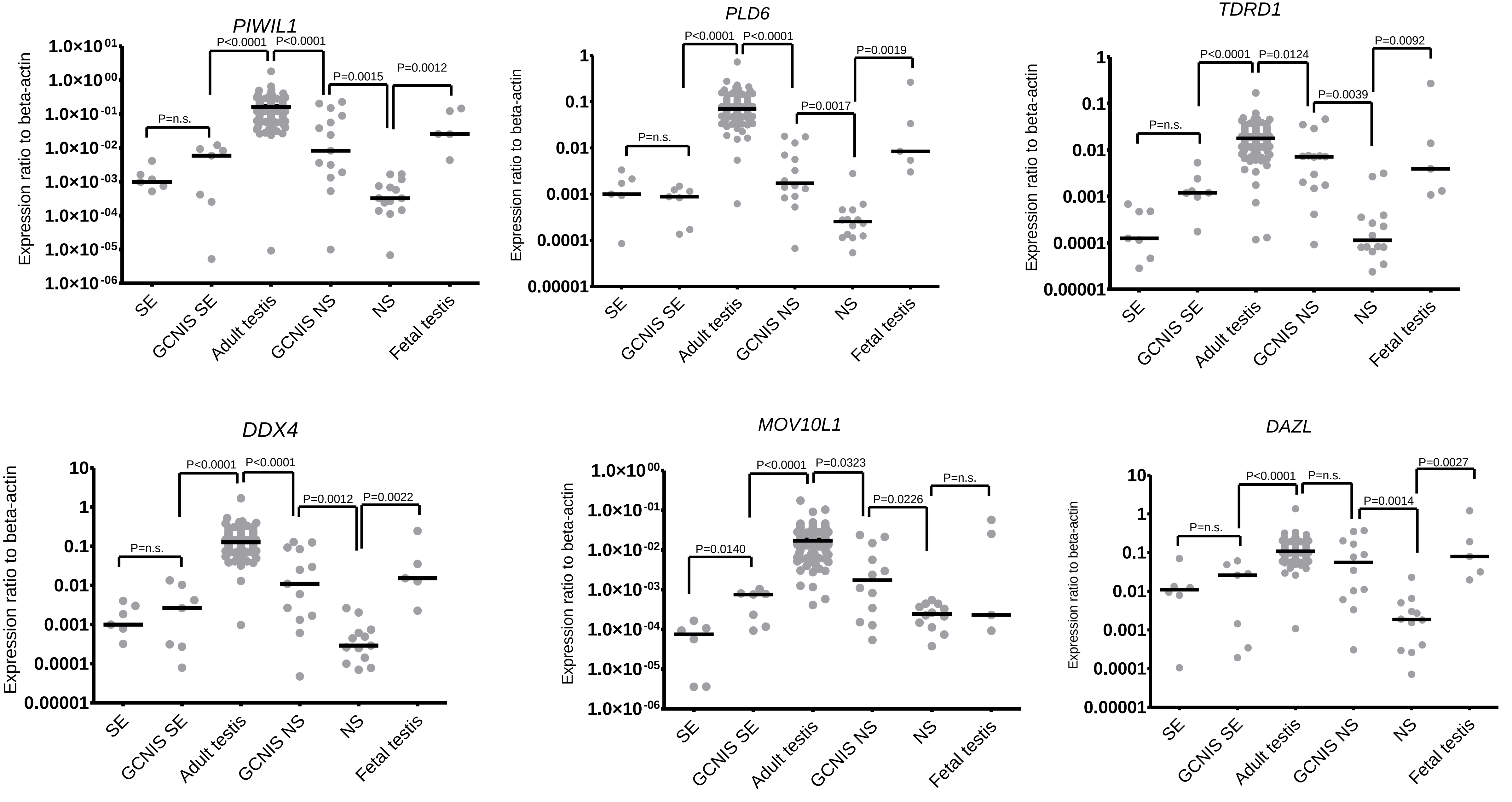

Supplementary figure S2. Expression level of TGCT/GCNIS markers in 6 pairs of testicular germ cell tumors (TGCTs) and their precursors GCNIS for 3 seminomas (SE) and 3 nonseminomas (NS). The charts also show data from publicly available datasets from fetal and adult healthy testis tissues (GTEx and supplementary table S9). P-values for two-tailed Mann-Whitney test are presented (n.s. – non-significant).

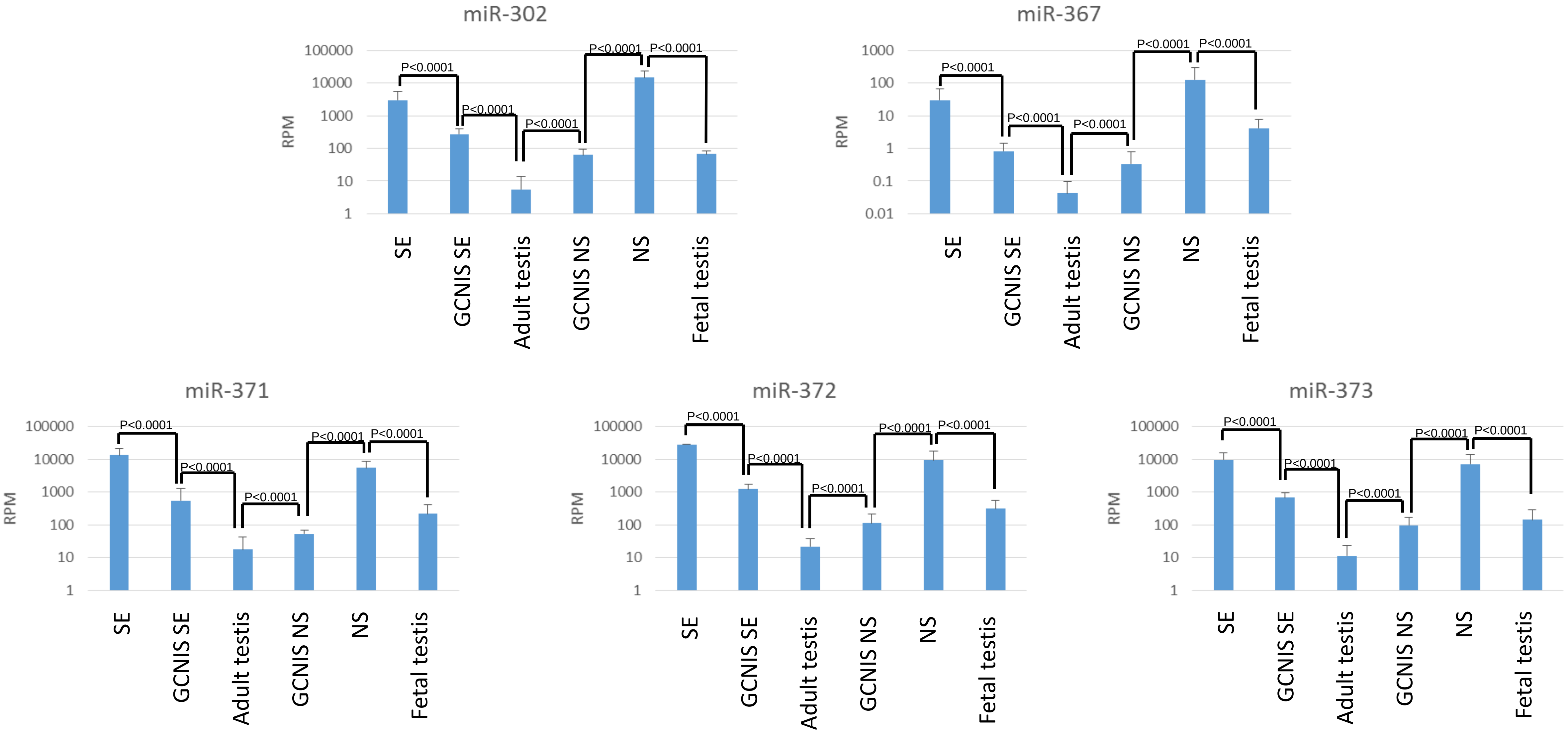

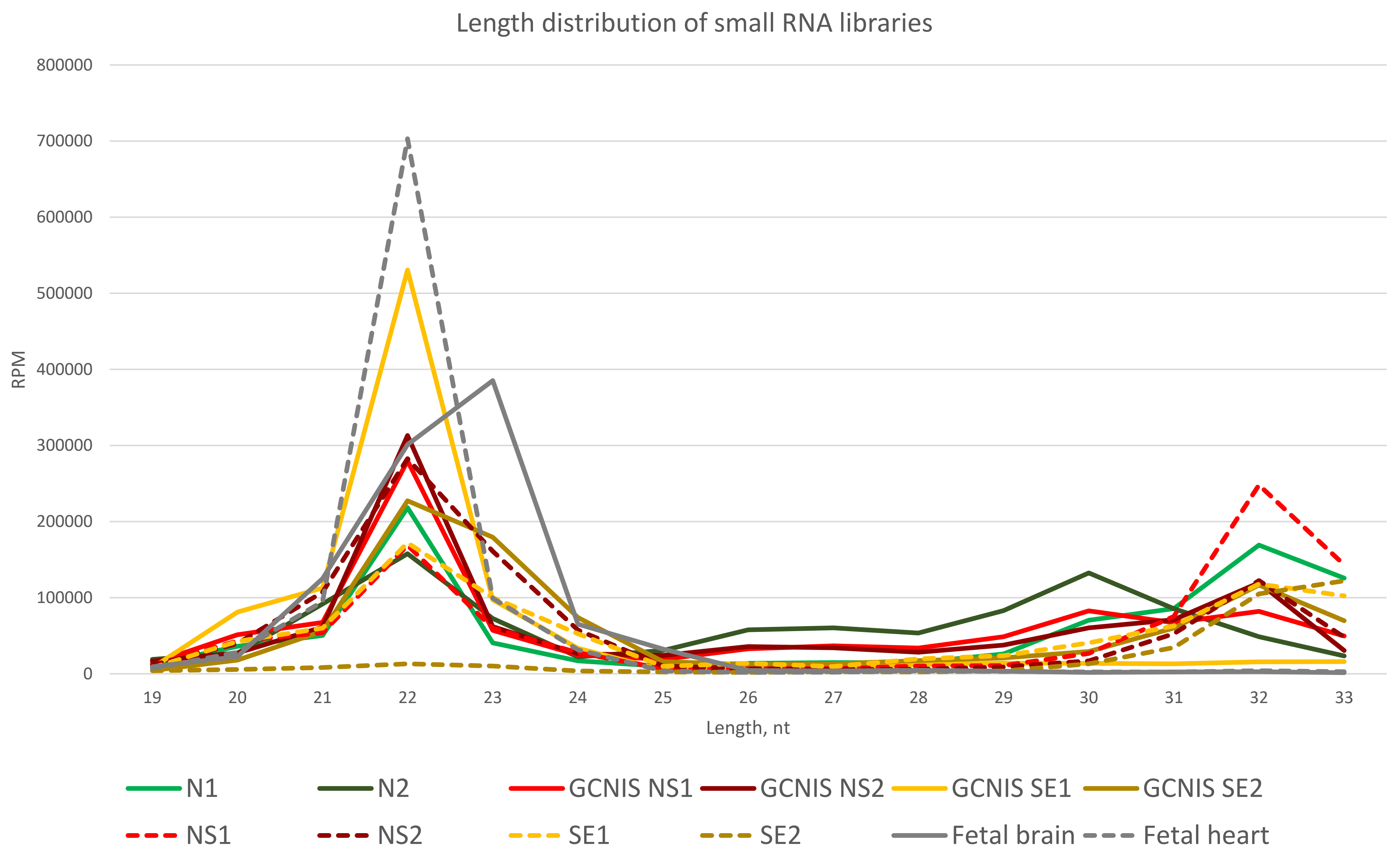

Supplementary figure S3. Length distribution of small RNA library reads in normal Adult testis (datasets N), TGCTs (SE for a seminoma and NS for nonseminoma) and matched adjacent GCNIS-containing testis tissues (GCNIS SE and GCNIS NS, respectively) as well as fetal somatic tissue.

Supplementary figure S4. Fraction of TE-deriving small RNAs in 6 pairs of testicular germ cell tumors (TGCTs) and their precursors GCNIS for 3 seminomas (SE) and 3 nonseminomas (NS) as well as normal fetal and adult testis tissues and somatic samples (supplementary table S9). P-values for two-tailed Mann-Whitney test are presented (n.s. – non-significant).

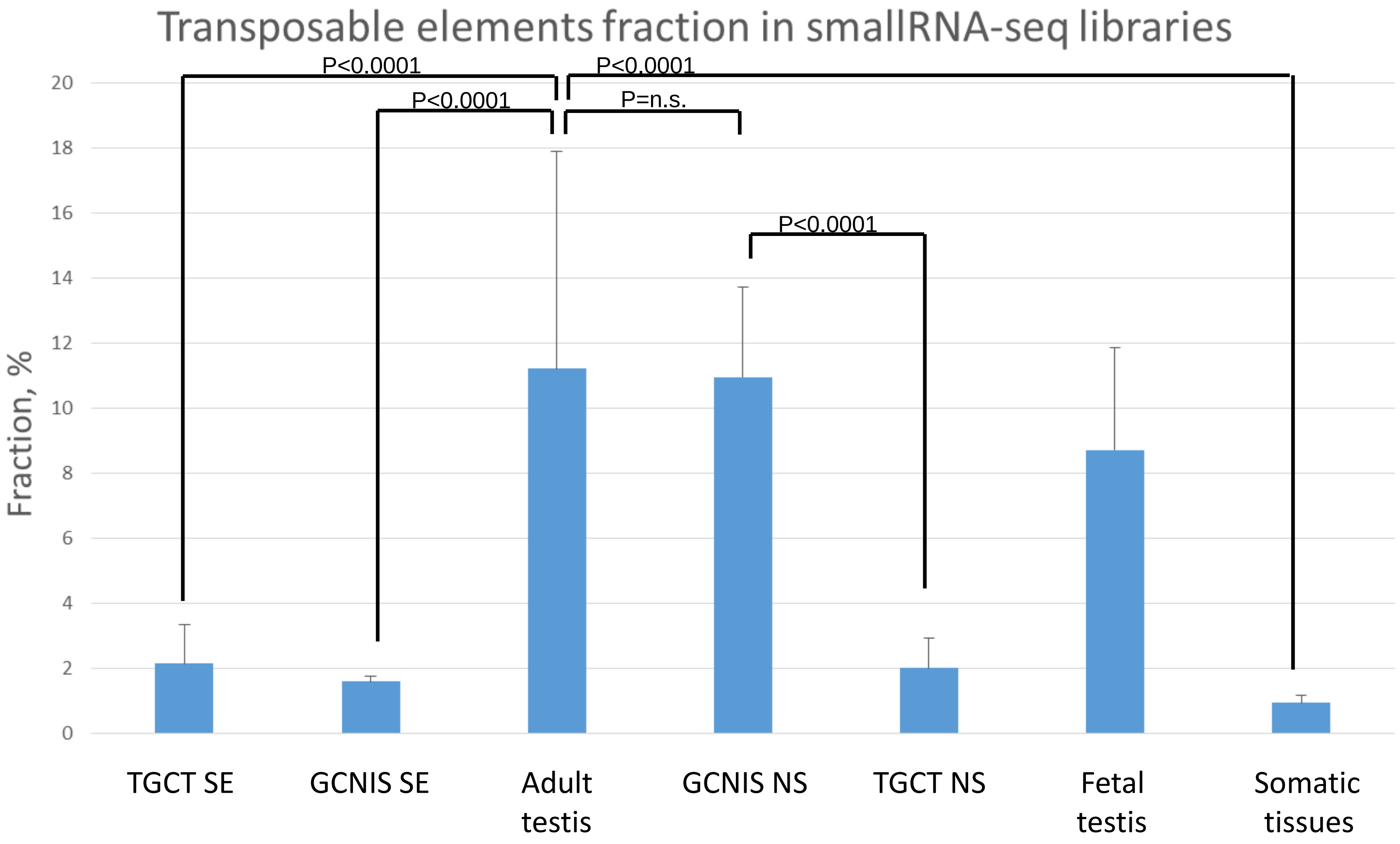

Supplement: Supplementary file 2 — (PDF 440 kb) [file 12885_2017_3945_MOESM2_ESM.pdf]
